# Transmission Dynamics of Methicillin-Resistant *Staphylococcus aureus* in Pigs

**DOI:** 10.3389/fmicb.2013.00057

**Published:** 2013-03-20

**Authors:** Florence Crombé, M. Angeles Argudín, Wannes Vanderhaeghen, Katleen Hermans, Freddy Haesebrouck, Patrick Butaye

**Affiliations:** ^1^Department of Bacterial Diseases, Veterinary and Agrochemical Research CentreBrussels, Belgium; ^2^Department of Pathology, Bacteriology and Avian Diseases, Faculty of Veterinary Medicine, Ghent UniversityGhent, Belgium

**Keywords:** MRSA, ST398, pigs, transmission risk factors, transmission routes, transmission pig models

## Abstract

From the mid-2000s on, numerous studies have shown that methicillin-resistant *Staphylococcus aureus* (MRSA), renowned as human pathogen, has a reservoir in pigs and other livestock. In Europe and North America, clonal complex (CC) 398 appears to be the predominant lineage involved. Especially worrisome is its capacity to contaminate humans in close contact with affected animals. Indeed, the typical multi-resistant phenotype of MRSA CC398 and its observed ability of easily acquiring genetic material suggests that MRSA CC398 strains with an increased virulence potential may emerge, for which few therapeutic options would remain. This questions the need to implement interventions to control the presence and spread of MRSA CC398 among pigs. MRSA CC398 shows a high but not fully understood transmission potential in the pig population and is able to persist within that population. Although direct contact is probably the main route for MRSA transmission between pigs, also environmental contamination, the presence of other livestock, the herd size, and farm management are factors that may be involved in the dissemination of MRSA CC398. The current review aims at summarizing the research that has so far been done on the transmission dynamics and risk factors for introduction and persistence of MRSA CC398 in farms.

## Introduction

*Staphylococcus aureus* is a major facultative pathogen, which is associated with a wide spectrum of diseases in both humans and animals (Mandell et al., 2000; Hermans et al., 2010). Ever since antimicrobial therapy was introduced, certain clones of this bacterium have shown the ability to gain resistance against almost all classes of antimicrobial agents to which they are exposed (Lowy, 2003; Malachowa and DeLeo, 2010). Of general concern is resistance to β-lactamase stable β-lactam antibiotics in methicillin-resistant *S. aureus* (MRSA). The first report on clinical cases of MRSA dates back to 1961, in the United Kingdom (Jevons, 1961). From then on nosocomial MRSA infections emerged, though infrequently, worldwide. In the late 1980s and the early 1990s, MRSA gradually increased in frequency and became a serious pathogen in hospitals throughout the world, the so-called healthcare or hospital-associated MRSA (HA-MRSA) (Enright et al., 2002; Grundmann et al., 2006). Still, in some countries of Europe (i.e., the Netherlands, Finland, Norway, Sweden, and Denmark), HA-MRSA infections have remained sporadic as a consequence of a strict Search and Destroy policy (Deurenberg et al., 2007). In the mid-1990s, a second wave appeared in the epidemiology of MRSA. Cases of MRSA were reported among people without healthcare-associated risk factors, now-called community-acquired MRSA (CA-MRSA) (Udo et al., 1993; Centers for Disease Control, and Prevention (CDC) (1999); Chambers, 2001; Okuma et al., 2002; Kluytmans-VandenBergh and Kluytmans, 2006). From then on, CA-MRSA emerged worldwide and became not only a threat in the community (with a low antimicrobial use) but also, occasionally, in the hospital environment (O’Brien et al., 1999; Saiman et al., 2003). CA-MRSA strains differ from HA-MRSA strains since they have a different accessory genome, carry different staphylococcal cassette chromosome *mec* (SCC*mec*) elements, affect different populations, and cause other clinical symptoms (Enright et al., 2002; Graffunder and Venezia, 2002; Grundmann et al., 2002; Okuma et al., 2002; Naimi et al., 2003; Robinson and Enright, 2003; Vandenesch et al., 2003; Ito et al., 2004; Tenover et al., 2006; Wijaya et al., 2006; Tacconelli et al., 2008; Witte, 2009; Yamamoto et al., 2010). Five clonal complexes (CCs), CC5, CC8, CC22, CC30, and CC45, were shown to prevail among HA-MRSA isolates while several genetic backgrounds (CC1, CC8, CC30, CC59, CC80, and CC93) were associated to the epidemic spread of CA-MRSA (Enright et al., 2002; Robinson and Enright, 2003; Vandenesch et al., 2003; Deurenberg and Stobberingh, 2009; Witte, 2009; David and Daum, 2010). Presently, however, it becomes ever more difficult to distinguish HA-MRSA from CA-MRSA (Song et al., 2011; Stefani et al., 2012), since clones with a typical hospital-acquired genetic background enter the community and typical clones with community-acquired genetic background enter the hospital (Campanile et al., 2011; Song et al., 2011).

In contrast to humans, antimicrobial susceptibility of *S. aureus* isolates in animals has been initially less continuously documented. The first isolation of MRSA was reported in 1972 from cases of bovine mastitis, with isolates that were believed to be from human origin (Devriese et al., 1972; Devriese and Hommez, 1975). Later on, MRSA was found occasionally in animals, mainly in pets and horses (Scott et al., 1988; Cefai et al., 1994; Hartmann et al., 1997; Lee, 2003; Goni et al., 2004). Here too, the strains were mostly human genotypes and accordingly, animals (and mainly companion animals) were perceived as potential vectors for (re-)infection of their human contacts (Scott et al., 1988; Cefai et al., 1994; Manian, 2003). However, in 2005, the first report of a new MRSA clone, in a pig farmer, initiated a third wave in the history of MRSA (Armand-Lefevre et al., 2005). Hereafter, Voss et al. (2005) reported MRSA in a family of pig farmers and their pig that appeared to be resistant to *Sma*I digestion, and thus not typeable by standard pulsed field gel electrophoresis (PFGE), and belonged to staphylococcal protein A gene (*spa*) type t108. The same MRSA type was isolated in two other cases including a pig farmer and a patient whose father was a veterinarian, which indicated a possible link between pig farming and an increased risk for MRSA carriage (Voss et al., 2005). Indeed, in an additional study, a 760-fold higher MRSA carriage rate among a group of regional pig farmers was found compared to the general Dutch population (Voss et al., 2005). These novel strains were typed using multilocus sequence typing (MLST) as sequence type (ST) 398 and, since the appearing of a ST variant, are generally grouped as CC398 (http://saureus.mlst.net). Since their discovery, livestock, and pigs particularly appeared to be an important reservoir for MRSA CC398 colonization and infection of humans in relation to farming worldwide (Huijsdens et al., 2006; Wulf et al., 2006, 2008a; van Loo et al., 2007; Witte et al., 2007; Khanna et al., 2008; Lewis et al., 2008; Nemati et al., 2008; Denis et al., 2009; Krziwanek et al., 2009; Persoons et al., 2009; Smith et al., 2009; Van den Eede et al., 2009; Mammina et al., 2010; Mulders et al., 2010; Graveland et al., 2011; Vandendriessche et al., 2011a; Crombé et al., 2012a). Moreover, a number of clinical cases caused by MRSA CC398 have been described in animals including pigs (Sergio et al., 2007; van Duijkeren et al., 2007; Schwarz et al., 2008; Meemken et al., 2010; van der Wolf et al., 2012), cows (Feßler et al., 2010; Vanderhaeghen et al., 2010; Holmes and Zadoks, 2011; Spohr et al., 2011), horses (Cuny et al., 2008, 2010; Hermans et al., 2008; Sieber et al., 2011), and dogs (Witte et al., 2007; Floras et al., 2010; Haenni et al., 2012). However, MRSA CC398 is not the only lineage recovered from pigs and other animals. Some types, such as ST9 and ST97, appear to be associated with livestock as well. Yet, MRSA ST398 and ST97 are mainly reported in Europe [European Food Safety Authority (EFSA), 2009; Battisti et al., 2010; Gómez-Sanz et al., 2010; Meemken et al., 2010] and the US (Smyth et al., 2009; O’Brien et al., 2012; Osadebe et al., 2012) while ST9 particularly prevails in Asian countries (Cui et al., 2009; Guardabassi et al., 2009; Neela et al., 2009; Wagenaar et al., 2009). Presently, MRSA strains originating from animals are commonly called livestock-associated MRSA (LA-MRSA). This review focuses on pigs as major reservoir of MRSA and on the possible transmission routes of LA-MRSA on pig herds and farms in general.

## Occurrence, Distribution, and Health Impact of LA-MRSA in Pig Herds

Since 2005, numerous publications have appeared focusing on the MRSA occurrence in pigs (Table 1). Most studies reported asymptomatic carriage of MRSA among pigs, in which CC398 appeared as dominant MRSA lineage, particularly in Europe. Currently, MRSA CC398 has been recognized in pigs or on pig farms in 18 European countries [European Food Safety Authority (EFSA), 2009; Huber et al., 2010; Habrun et al., 2011; Overesch et al., 2011], on the American continent in Canada (Khanna et al., 2008), the USA (Smith et al., 2009; Molla et al., 2012; Osadebe et al., 2012; O’Brien et al., 2012) and Peru (Arriola et al., 2011), and also in Asia, including Singapore (Sergio et al., 2007), China (Wagenaar et al., 2009), and Korea (Lim et al., 2012). Furthermore, a wide variety of non-CC398 MRSA types have been identified in pigs or on pig farms (Table 1). In Asia, MRSA CC9 appears as the most prevalent clone associated with pig farming (Sergio et al., 2007; Cui et al., 2009; Guardabassi et al., 2009; Neela et al., 2009; Wagenaar et al., 2009; Larsen et al., 2012; Lo et al., 2012; Tsai et al., 2012; Vestergaard et al., 2012). However, MRSA strains with a “typical” human genetic background (ST5, ST8, ST22, ST30, and ST45) have also been reported in Europe, USA, and Africa, which might indicate transmission of (human) MRSA strains from humans to pigs (Sergio et al., 2007; Khanna et al., 2008; Pomba et al., 2009; Overesch et al., 2011; Fall et al., 2012; Molla et al., 2012; O’Brien et al., 2012).

**Table 1:** **Summarized chronology of publications reporting MRSA carriage and infection in pigs and their human contacts, 2005–2012**.

Year of study	Location	Major finding(s)	Genotype(s) identified^a^	Reference
NS	France	New MRSA clone ST398 in pig farmers. 4.5% (5/112) pig farmers carried MRSA in the nasopharynx; none of the 27 non-farmers matched by age, sex, and country of residence carried MRSA. No MRSA results from owners’ pigs	ST8, ST5, ST438, and ST398	Armand-Lefevre et al. (2005)
2004–2005	Netherlands	Association between pig farming and high MRSA carriage rates. Three family members (including a 6-month-old girl, patient A) on a pig farm (A) carried identical MRSA strains; another farmer (patient B), a veterinarians’ son (patient C), his father, and his nurse carried the same strain as members of farm A; 3.3% (1/30) pigs on farm A had perineal carriage of the same strain; at a meeting of regional pig farmers, 23% (6/26) were colonized with MRSA in the throat and/or the nose	NT by *Sma*I PFGE; *spa* type t108, t567, or t943	Voss et al. (2005)
2005	Netherlands	Clonal spread of MRSA ST398 and transmission between humans and pigs. A woman with MRSA mastitis and her daughter had MRSA nasal colonization; three family members and three co-workers had MRSA throat or nasal carriage; 80% (8/10) pigs had throat, nasal, or perineal carriage	ST398/t108; *agr* type 1; PVL−; TSST-	Huijsdens et al. (2006)
2005	Singapore	3.1% (2/64) pigs used in experimental research, 2% (1/50) pigs in a slaughterhouse, and 2% (1/29) staff workers at an academic hospital’s research facilities had nasal MRSA colonization	Pig isolates: ST398-V; pig and human isolates: ST22-IV	Sergio et al. (2007)
2005	Denmark	*S. aureus* nasal carriage in 10% (10/100) slaughter pigs; 10% (1/10) were MRSA, and 90% MSSA	NT by *Sma*I PFGE; *spa* types t034 and t1793	Guardabassi et al. (2007)
2005–2006	Netherlands	39% (209/540) pigs in nine slaughterhouses carried MRSA in the nares; transmission of MRSA both prior to arrival and at slaughterhouse was likely	ST398-III^b^, IVa or V/t011, t108, t1254, t1255, t567, t034, and t943	de Neeling et al. (2007)
2006	Netherlands	Purchase of MRSA-positive pigs as transmission route for MRSA spread. MRSA SSTI in 4 piglets on a breeding farm and 20 pigs on a supplier farm; MRSA nasal carriage in 2 farm workers	ST398-IV/t011	van Duijkeren et al. (2007)
2006	Netherlands	Transmission of MRSA ST398 between different kinds of pig farms through purchase of MRSA-positive pigs. 11% (35/310) pigs on 23% (7/31) farms had MRSA nasal colonization; 11 MRSA-positive personnel had strains with identical genotype as those of the pigs of their respective farms	ST398-IV or V/t011, t108, t899, and t1939; PVL−, TSST-	van Duijkeren et al. (2008)
2004–2007	Denmark	Pigs as a source of MRSA CC398. Pigs tested after a person working or living on a pig farm presented with MRSA CC398, *spa* types t034, t108, or t1793, infection or carriage; 46% (23/50) pigs on 80% (4/5) farms had nasal carriage of MRSA CC398	Pig isolates: CC398/t034	Lewis et al. (2008)
2007	Germany	Strong association between in-herd prevalence and pig contact intensity. 13% (85/678) pigs from 18% (62/347) farms were MRSA-positive; 23% (20/86) human contacts carried MRSA	ST398	Meemken et al. (2008)
2007	Germany	Pigs are a reservoir for import of MRSA in hospitals. MRSA was isolated on 70% (28/40) of the farms; no pig colonization rate since nasal samples were pooled	ST398-IV or V/t011, t108, t1451, t2510; PVL−; TSST-	Köck et al. (2009)
2007	Netherlands	Working with pigs is a high risk for acquiring MRSA. 56% (28/50) farms were MRSA-positive with MRSA detected in pigs or dust samples; 30% (15/50) farms had one or more MRSA-positive persons	NT by *Sma*I PFGE; Pig isolates: t011, t108, t567, t899, t2330; human isolates: t011, t108, t567, t588, t2330, t2741	van den Broek et al. (2009)
2007	Italy	A farm worker with clinical symptoms was infected with MRSA ST398; 9.1% (1/11) people living or working on the farm were MRSA-positive	Patient: ST398-IVa/t899; Co-worker: *spa* type t108, SCC*mec* type V	Pan et al. (2009)
NS	Ontario, Canada	MRSA with identical genotype among pigs and humans. 24.9% (71/285) pigs on 20 farms had MRSA nasal or rectal colonization; 20% (5/20) pig farmers had MRSA nasal carriage; on five farms with human colonization, concordant strain types were found in farmers and pigs	Pig and human isolates: *spa* type t034 and NT by *Sma*I PFGE; pig and human isolates: USA100-CC5/t002	Khanna et al. (2008)
2007	Belgium	66.3% (273/412) pigs were MRSA-positive (nares, skin, perineum, or rectal samples) on 2 MRSA-positive farms; people living on one of the two farms had nasal MRSA colonization	NT by *Sma*I PFGE	Dewaele et al. (2011)
2007	Belgium	44% (663/1500) pigs belonging to the 68% (34/50) of the farms sampled carried MRSA.	ST398-IVa or -V/t011, t034, t567, and t2970	Crombé et al. (2012a)
2003–2008	Netherlands	MRSA from post-mortem samples from pigs. 16% (19/116 pigs with *S. aureus*) isolates were MRSA, with MRSA being the first cause of infection in 11 pigs	CC398/t011, t108, t367, t899 and t2330	van der Wolf et al. (2012)
2007–2008	Iowa and Illinois, USA	Pigs as important reservoir of MRSA ST398. In two farm systems, 49% (147/299) swine and 45% (9/20) farm workers had MRSA nasal carriage	Pigs and workers isolates: ST398-V; PVL−	Smith et al. (2009)
2008	China	MRSA from Chinese pigs differ from European LA-MRSA clone. MRSA isolated from dust samples on 5/9 (56%) pig farms in Sichuan Province	ST9/t899; ST1376/t899; PVL−	Wagenaar et al. (2009)
2008	China	MRSA from Chinese pigs and farm workers differ from European LA-MRSA clone. MRSA isolates from nares of 11.4% (58/509) pigs and 15% (2/13) farm workers in four Chinese provinces	ST9/t899; ST912/t899; ST1297/t899; PVL−	Cui et al. (2009)
2008	Portugal	Four pigs and one veterinarian from a pig farm had MRSA nasal carriage and at a second farm, three pigs had MRSA nasal carriage	Farm 1: ST398-V/t011, PVL−; Farm 2: ST30-V/t021, PVL−	Pomba et al. (2009)
NS	Malaysia	Low prevalence of MRSA in pigs. One or more pigs had MRSA nasal carriage on 30% (9/30) of the farms; 5.5% (5/90) humans in contact with pigs had MRSA nasal carriage	ST9-V/t4358; ST1-V/t1784	Neela et al. (2009)
2008	Hong-Kong	16% (16/100) carcasses on two wet markets had nasal MRSA colonization. No possibility to access to living pigs	ST9-IVb or V/t899	Guardabassi et al. (2009)
NS	Germany	Study1: 70.8% (368/520) slaughter pigs from 4 abattoirs; Study 2: 49% (248/506) slaughter pigs from 1 abattoir had nasal MRSA colonization	ST398-V or III^b^/t011 and t034	Tenhagen et al. (2009)
2008	Europe	MRSA ST398 is widely distributed throughout Europe. 11.7% pig breeding holdings and 25.5% pig production holdings are MRSA ST398-positive. *Results are based on dust samples following the EFSA guidelines*	Dominant clone: ST398/t011; CC1; CC5; CC8;CC9; ST39 (CC30); CC97; ST132 (CC133)/multiple *spa* types	European Food Safety Authority (EFSA) (2009)
2008	Italy	Heterogeneity among MRSA in finishing pigs. 14% (98/701) pools (10 pigs/pool and 6 pools/farm) were MRSA-positive on 38% (45/118) positive holdings	ST398/t011, t034, t108, t899, t2510, and t2922; ST1/t127; ST1476/t1730; SCC*mec* type V, IVb or 2B + 5^d^; ST9-V/t4794; ST97-V/t4795; ST398-2B + 5^d^/t4838	Battisti et al. (2010)
2008	Germany	52% (152/290) fattening pig farms are MRSA-positive; with a prevalence from 39% to 59% from east to south-west of the country. *Results are based on dust samples*	CC398-V, V^c^, Iva, or NT/t011, and t034	Alt et al. (2011)
2007–2008	Spain	MRSA carriage is lower in Iberian pigs (28%, 30/106;) than in Standard White pigs (83%, 130/157)	ST398/t011, t034; ST1966/t011, ST1968/t011; ST1969/t011	Porrero et al. (2012)
2007–2009	Ireland	Absence of MRSA CC398 in pigs and humans. 0% (0/440) pigs from 41 farms had MRSA-positive nasal samples; 2% (2/101) human contacts carried (human) MRSA strains	ST22; ST1307	Horgan et al. (2011)
2008–2009	Korea	MRSA clones from both animal and human origin are distributed among pigs. 3.2% (21/657) pigs carried nasal MRSA on 22.7% (15/66) of the farms	ST398/t034; ST541/t034; ST72/t664, and t2461	Lim et al. (2012)
2008–2009	Spain	Pig-to-human transfer of MRSA ST398. MRSA-positive pig farmer with skin lesion; 91.7% (11/12) pigs had nasal MRSA colonization	Patient and pig isolates: ST398-IVa or V/t011 and t108; Patient isolate: ST398-V/t588	Lozano et al. (2011a)
2008–2009	USA	1.3% (2/157) samples from pigs exhibited at shows were MRSA-positive	ST398/t3075; ST2136 (CC9)/t337	Dressler et al. (2012)
2009–2010	Switzerland	Increase of MRSA prevalence within 2 years among slaughter pigs. 2% (8/405) slaughter pigs had MRSA nasal colonization; 1 year later 5.9% (23/392) had MRSA nasal colonization	ST398-V/t034, t011, and t1451; ST49-V/t208; ST1-IVc/t2279	Overesch et al. (2011)
2009	Denmark	74% (230/311) pigs had MRSA nasal carriage on 6 MRSA-positive farms	CC398-V/t011 and t034	Espinosa-Gongora et al. (2011)
2009	Switzerland	1.3% (10/800) pigs carried nasal MRSA; no MRSA among 148 pig farmers attending meetings on swine breeding	ST398-V/t034	Huber et al. (2010)
NS	Spain	Other MRSA lineages than CC398 are able to spread among pigs. 20.8% (11/53) finishing pigs and 49.1% (26/53) suckling pigs coming from two abattoirs (six production chains) had nasal MRSA colonization	ST398/t011, t108, t1197, and t2346; ST1379/t3992 (CC97)	Gómez-Sanz et al. (2010)
NS	Serbia	7.1% (6/84) pigs had nasal MRSA colonization	CC45-IVa/t015	Velebit et al. (2010)
2009	Peru	40% (8/20) pigs had nasal MRSA carriage originating from one out of six large-scale holdings; 5% (1/20) scavenging pigs had nasal MRSA carriage originating from 1 out of 6 rural communities	ST398-V/t571; USA300-like ST8-IVa/t008	Arriola et al. (2011)
2009	Denmark	13% (101/789) of pigs at slaughter have MRSA, with 93% of MRSA belonging to CC398, 4% to CC30, and the remaining to CC1, CC30 isolates carried SCC*mec* V + cadmium zinc resistance gene *czrC*, meaning spread of typical CC398 SCC*mec* to other lineages	CC398/t011, t034, t1451, t2876, t2974; CC30-V + *czrC*/t1333; CC1/t0127	Agersø et al. (2012)
2009	Belgium	In 26 of 30 farms (pig and mixed farms), pigs carried MRSA, No effect of the farm type (pigs only or multispecies) on the MRSA status of the pigs	ST398-IVa and -V/t011, t034, t567, t571, t1451, t2974, t3423, and t5943	Verhegghe et al. (2012b)
2009–2011	Dakar	1.3% (6/464) pigs positive for MRSA	ST5-IV/PVL+; ST88-IV	Fall et al. (2012)
2009–2010	Spain	85.7% of 300 pigs analyzed and 9.3% of 54 pig workers screened carried CC398 MRSA	ST398-IV or V	Morcillo et al. (2012)
2009–2010	Netherlands	3.2% (11/341) pig slaughterhouse workers, 47% (40/85) gloves samples, and 27.5% (11/40) air samples were MRSA-positive	ST398/t011, t064, t108, and t2330; ST7/t091; ST8/t064; ST45/t015	Gilbert et al. (2012)
2009–2010	Taiwan	42.5% (127/299) pigs from 11 counties in western Taiwan carried MRSA. 220 MRSA isolates were recovered from the 127 positive pigs. 36 pigs (28.3%, 36/127) harbored more than one MRSA strain	ST9-IV o V/t899 t1939, t2922, t4132, t4358, and t7616; PVL-	Lo et al. (2012)
2010	Spain	Pig-to-human transfer of MRSA ST398. MRSA-positive pig farmer with skin lesion; 50% (9/18) pigs had nasal MRSA	ST398-V/t011 and t1451	Lozano et al. (2011b)
2010	Iowa, Minnesota, and New Jersey, USA	58.2% (230/395) with MSSA and 6.6% (26/395) pork samples with MRSA	23.1% MRSA CC398/t011 and t034; CC5/t002; CC8/t008, and other *spa* types (t094, t078, t273, t803, t2922, t8314)	O’Brien et al. (2012)
2010	Netherlands and Germany	Absence from pig contact during the summer leave did not have an impact on MRSA colonization of pig farmers. 9% of the farmers lost MRSA during summer leave	t011, t034, t108, t1451, t1197	Köck et al. (2012)
2010	Connecticut, USA	3% (8/263) pigs and 22% (2/9) humans carried MRSA on 14% (5/35) farms	12.5% (1/8) ST398/t034, PVL+; 50% (4/8) MRSA USA300/t008, PVL+; 12.5% MRSA USA 200/t007, PVL+; 25% PFGE NS/t007, PVL- and PVL+	Osadebe et al. (2012)
2011	Thailand	MRSA-positive in 15% of the 20 small-scale and none of the 10 large-scale confined production holdings	ST9-IX/t337	Larsen et al. (2012)
2011	Thailand	4% (5/126 pig samples) MRSA isolates	ST9/t899	Tsai et al. (2012)
2011	Thailand	50% (5/10) pork samples and 40% (6/15) pig nasal swabs positive for MRSA	CC9 (ST9, ST2136, ST2278)-IX/t337	Vestergaard et al. (2012)
NS	Thailand	10% (4/40) weaning pigs had nasal MRSA colonization	ST9/t337, PVL-, TSST-	Anukool et al. (2011)
NS	Croatia	35.3% (24/68) samples were MRSA-positive obtained from 8 large pig breeding farms – *Results are based on dust samples*	t011, t108, and t1451	Habrun et al. (2011)
NS	Denmark	72.6% (284/391) samples with MRSA CC398, including 230 (74%) animal and 54 (68%) environmental samples (dust samples) at six Danish-MRSA-positive farms. PFGE analysis revealed the existence of farm-specific pulsotypes, spread of MRSA CC398 in Danish pig farms is mainly due to clonal dissemination of farm-specific lineages	ST398-V/t011, t034	Espinosa-Gongora et al. (2012)
NS	Germany	Absence of MRSA CC398 in alternative farms. In farms practising alternative farming different from the intensive common farming practices, no *S. aureus* isolates were obtained from nasal swabs from pigs (178 animals analyzed). 34.8% (31/89) nasal human samples were positive for MSSA not CC398. The only person carrying MRSA CC398 had worked in an intensive farm	CC1, CC5, CC8, CC9, CC15, CC25, CC30, CC34, CC45, CC59, CC97, CC121, CC398, ST426	Cuny et al. (2012)
NS	Ohio, USA	3% (7/240) pigs sampled on farm before marketing, 11% (27/240) holding pens at the slaughterhouse, 2% (4/235) of carcasses, and 4% (5/135) of retail pork samples were MRSA-positive	ST398/t034, t539 and t1435; ST5/t002 and t268; ST9/t1435; ST39/t123; ST72/t049; ST1340/t002	Molla et al. (2012)

*^a^*S. aureus* strain genotypes are presented in the following format: multilocus sequence type (MLST) or Clonal Complex (CC)-SCC*mec* type/*spa* type*.

*^b^SCC*mec* type using the method of Zhang et al. (2005)*.

*^c^SCC*mec* subtype Vc*.

*^d^SCC*mec* type 2B + 5 (*ccrA2B2*-*mecB* + *ccrC*) using the method of Kondo et al. (2007)*.

*LA-MRSA, livestock-associated methicillin-resistant *S. aureus*; MRSA, methicillin-resistant *S. aureus;* MSSA, methicillin-susceptible *S. aureus*; NS, Not supplied; NT, not typeable; PFGE, Pulse field Gel Electrophoresis; PVL, presence (+) or absence (−) of the Panton-Valentine leukocidin; SSTI, skin, and soft tissue infection; TSST, presence (+) or absence (−) of the toxic shock syndrome toxin*.

In 2008, a European baseline study determined the MRSA prevalence in both breeding (i.e., housing and selling breeding pigs) and production holdings (i.e., housing breeding pigs and selling pigs for fattening or slaughtering) from 24 European Union member states and 2 non-member states, based on the analysis of environmental samples [European Food Safety Authority (EFSA), 2009]. Both production types were distinguished since breeding holdings are generally considered to have a better status in terms of management and hygiene practices, health status, and biosecurity measures [European Food Safety Authority (EFSA), 2009; European Food Safety Authority (EFSA), 2010]. The reported MRSA CC398 prevalence varied significantly between countries at breeding holding level, ranging from 0% in 14 states to 46% in Spain, and at production holding level, ranging from 0% in 11 states to 50.2% in Spain. However, pooling of environmental wipes probably resulted in substantial underestimation of the true prevalence, especially on farms with low in-herd prevalence (Broens et al., 2011a). In the Netherlands, for example, Broens et al. (2011b) reported a MRSA-positive herd prevalence of 67.3% in breeding holdings and 71.0% in finishing holdings while in the European baseline study, the herd prevalence was 12.8 and 17.9%, respectively [European Food Safety Authority (EFSA), 2009]. In addition, farm level rates may increase over time (Broens et al., 2011b; Overesch et al., 2011). Broens et al. (2011b) reported an increase from ∼30 to 75% over a 2-year time-period, probably as a consequence of MRSA transmission between herds.

At animal level the prevalence also differs considerably between countries, ranging from 10% in Denmark (Guardabassi et al., 2007) to 44% in Belgium (Crombé et al., 2012a). Moreover, age-related differences in MRSA prevalence were reported (Smith et al., 2009; Broens et al., 2011b; Weese et al., 2011; Crombé et al., 2012a). Piglets have manifestly higher carriage rates compared to sows and fattening pigs (Smith et al., 2009; Broens et al., 2011b; Weese et al., 2011; Crombé et al., 2012a). Also, differences between breeds of pigs have been reported. Indeed, MRSA carriage appeared lower in Iberian pigs than in standard white pigs (Porrero et al., 2012). However, this might also be due to differences in rearing methods with Iberian pigs being more outdoors. Hence, caution must be taken when comparing results of between-herd and within-herd prevalence from various studies due to differences in sampling and isolation procedures, number of pigs sampled, sample size and sample populations (finishing vs. breeding pigs, piglets vs. older pigs, open vs. closed farms, pigs at the abattoir vs. pigs at the farm, etc.) (Broens et al., 2011a).

Despite the high MRSA carriage rates, clinical MRSA infections are rarely reported in pigs. MRSA CC398 has been associated with a case of exudative epidermitis in 2006, a condition typically due to *Staphylococcus hyicus* (van Duijkeren et al., 2007). In addition to skin infections, sporadic reports of infections of the urogenital tract, the uterus, and mammary gland as well as from deep-seated tissues and septicemia have been described (Schwarz et al., 2008; Kadlec et al., 2009; Meemken et al., 2010). Meemken et al. (2010) reported that 43% (60/138) of the *S. aureus* isolates originating from pathological lesions in pigs, collected over a 4-years period, were identified as MRSA; of these, 95% (57/60) were MRSA CC398, with the remaining being MRSA CC97. In 35% (21/60) of the cases, MRSA could be considered as primary causative agent for the identified lesion. In another study, *S. aureus* [including methicillin-susceptible *S. aureus* (MSSA) and MRSA] was only isolated from ∼0.6% (*n* = 144) of the pig samples submitted for post-mortem examination over a 6-years period (van der Wolf et al., 2012). From these *S. aureus*-positive cases, 116 were included in the study for further investigation. *S. aureus* was recognized as primary cause of infection in 62% (72/116) of the cases. Of these, 16.3% (19/116) were MRSA of which 52% (11/19) assigned MRSA as primary cause of infection. Eighteen strains belonged to CC398 and one to non-CC398 ST1, *spa* type t127. According to these results, the impact of MRSA on pig health is relatively minor at the moment.

## Risk Factors for Introduction and Persistence of LA-MRSA in Pig Herds

The European baseline study showed that some factors may be associated with MRSA contamination of breeding holdings, namely herd type, herd size and gilt, and boar replacement policy [European Food Safety Authority (EFSA), 2010]. Accordingly, van Duijkeren et al. (2008) reported that 83.3% (5/6) of the investigated herds, supplying pigs to MRSA-positive herds, were MRSA-positive. Moreover, Broens et al. (2011c) reported an 11-fold higher odds ratio for herds, with a MRSA-positive supplier, to be MRSA-positive. Hence, animal trading appears to be an important factor for introduction of MRSA on MRSA-negative herds [van Duijkeren et al., 2008; European Food Safety Authority (EFSA), 2009; Broens et al., 2011c]. Yet, additional risk factors are implicated. Indeed, Broens et al. (2011c) found that 23% of the herds with a negative supplier and 46% of farms without supplier were MRSA-positive.

Broens et al. (2011b) conducted a risk factor analysis in the Netherlands and reported, in accordance with the European baseline study, that herd size was highly associated with MRSA prevalence. Larger herds appear more likely to be MRSA-positive compared to smaller herds, due to a higher risk of introduction (between-herd dynamics), a higher number of susceptible animals by birth or purchase, and a higher probability of persistence in larger herds (within-herd dynamics) (Broens et al., 2011b). In this study, however, each individual management variable (i.e., purchase of gilts, hygiene score, and antimicrobial use) was too small to yield a significant effect on the MRSA prevalence but still a significant association was observed between each variable and herd size (Broens et al., 2011b). Consequently, larger herds have a higher probability to be MRSA-positive since multiple risk factors (antimicrobial use, animal trade, and low hygiene level) affecting MRSA prevalence are positively associated with herd size (Broens et al., 2011b). In another risk factor study, performed in Germany, herd type, and herd size were shown to play a crucial role in dissemination of MRSA in fattening holdings (Alt et al., 2011). Also regions, type of floor, purchase of pigs, antimicrobial use, and presence of cattle on the farm and animal-flow system were associated with a positive MRSA test result (Alt et al., 2011). However, further research is still necessary to investigate the role of additional factors involved in the dissemination of MRSA on pig farms since MRSA has also been reported on closed farms implementing more stringent biosecurity measures [European Food Safety Authority (EFSA), 2009; Alt et al., 2011]. For example, airborne transmission in areas with a high density of pig farms might be involved in the spread of MRSA between farms given that MRSA has already been reported outside MRSA-positive farms to 150 m downwind (Schulz et al., 2012). Also, it is clear that the environment plays a role in the transmission of MRSA in farms, since similar CC398 clones have been found among farmers, animals, and environmental samples (Espinosa-Gongora et al., 2012; Pletinckx et al., 2012).

Antimicrobial use is a factor that deserves special attention, as it is an obvious factor suggested to be associated with the emergence and spread of MRSA (Tacconelli et al., 2008; van Duijkeren et al., 2008; Kadlec et al., 2012). However, so far, no straightforward relationship appears from the literature. Broens et al. (2011b,c) reported that batch treatments with antimicrobials resulted in a higher prevalence, though not significant, compared with batches that were not subjected to these treatments. In another longitudinal field study, higher transmission rates were observed when tetracycline and β-lactams were used (Broens et al., 2012b). In addition, feed supplemented with tetracycline appeared to increase the nasal MRSA CC398 load of piglets in an experimental study (Moodley et al., 2011a). Tetracycline resistance is independent of the SCC*mec* (Aarestrup et al., 2010), which contains the methicillin resistance gene (*mecA*), although the SCC*mec* cassette type III has a integrated copy of the plasmid pT181 with the tetracycline resistance gene *tet*(K) (Jensen and Lyon, 2009; Turlej et al., 2011). The use of tetracycline may play a role in the selection and increase of transmission rates of ST398 isolates, since tetracycline resistance genes are present in nearly all ST398 (both MRSA and MSSA) isolates. This broad spread of tetracycline resistance genes has probably been promoted by the use of tetracycline in pig farming, as this antibiotic is one of the most prescribed antibiotics for pigs (Anonymous, 2008; Callens et al., 2012). In fact, only few tetracycline susceptible ST398 strains have been isolated (Davies et al., 2011; Zarfel et al., 2012). But, apart from tetracycline use, the use of other antimicrobial agents could promote the emergence of MRSA CC398. Recently, MRSA ST398 with decreased susceptibility to tiamulin, a pleuromutilin antimicrobial used exclusively in veterinary medicine, has been reported (Kadlec et al., 2010; Rubin et al., 2011). This fact deserves further attention since several genes responsible for pleuromutilin resistance have been found in CC398 isolates (Kadlec and Schwarz, 2009; Kehrenberg et al., 2009; Kadlec et al., 2010; Mendes et al., 2011; Schwendener and Perreten, 2011; Lozano et al., 2012). The first pleuromutilin resistance gene reported in CC398 was *vga*(C), which also confers resistance to lincosamides and streptogramin A (Kadlec and Schwarz, 2009). This gene is located on a multiresistance plasmid which carries antimicrobial resistance genes *aad*(D), *tet*(L), and *dfr*(K) as well (Kadlec and Schwarz, 2009). More recently this *vga*(C) gene has also been found in a small plasmid (Kadlec et al., 2010). Later on, other *vga* genes were reported among CC398 isolates including the *vga*(A) gene carried in different plasmids (Mendes et al., 2011; Lozano et al., 2012), the *vga*(A) variant *vga*(A)v, and *vga*(E), both chromosomal located on different transposons (Schwendener and Perreten, 2011; Lozano et al., 2012). An additional pleuromutilin resistance gene found among CC398 is the gene *cfr*, which also confers resistance to phenicols, streptogramin A and oxazolidinones (Kehrenberg et al., 2009). The *cfr* gene is located on plasmids and is transferable within and between staphylococcal species. Also, it was first detected in a plasmid from a bovine *Staphylococcus sciuri* strain (Kadlec et al., 2012). Especially worrisome is that this multiresistance gene has also been found in other gram-positive and gram-negative bacteria (Kadlec et al., 2012). Although these data suggest that various antimicrobial agents play a role in the ST398 transmission, there are some studies that report high transmission rates without the use of antimicrobial treatment. Indeed, Crombé et al. (2012b) have shown an extremely efficient transmission of MRSA CC398 between pigs without any antimicrobial treatment. It has even been shown that MRSA can be present in pigs with no antimicrobial use at all (Weese et al., 2011). On the other hand, in alternative pig farming systems, where no preventive antimicrobial treatment is used, absence of MRSA was reported (Cuny et al., 2012). Nevertheless, such loose data are difficult to interpret since more factors differ between these organic farms and conventional pig farms. The alternative farming, as studied by Cuny et al. (2012), in contrast to conventional fattening methods implies smaller farms with straw bedding and low animal density. Interestingly, in this study, one farm worker, who previously worked in a conventional pig farm, carried MRSA CC398 (Cuny et al., 2012). Consequently, it seems that antimicrobial use is not requested for MRSA acquisition and transmission among pigs but it is likely to have some influence on the MRSA load and/or to predispose animals to MRSA colonization, which might result in an increased prevalence at farm level.

Besides antimicrobials, heavy metals such as copper have been shown to promote co-selection of antimicrobial resistance and probably the spread of antibiotic resistant bacteria (Hasman et al., 2006). In fact, zinc-oxide appeared to increase the nasal MRSA CC398 load of piglets (Moodley et al., 2011a). Zinc is commonly used in pig feed, at 50–125 ppm, as it plays an important role in various physiological processes (Katouli et al., 1999; Hill et al., 2000). Moreover, zinc fed at high dietary levels (2000–3000 ppm) is widely used in the early phases of the nursery period since it reduces the incidence of diarrhea and increases weight gain in newly weaned pigs (Jacela et al., 2010). Hence, it has been hypothesized that the emergence of MRSA ST398 in pigs is also driven by the use of zinc-oxide (de Neeling et al., 2007; van Duijkeren et al., 2008; Aarestrup et al., 2010; Cavaco et al., 2011). Zinc-oxide has the potential to co-select specifically for MRSA ST398 since *czrC*, which encodes for cadmium and zinc resistance, is present within the SCC*mec* cassette type V element in ST398 (Cavaco et al., 2010). Notify, however, that a substantial proportion of MRSA ST398 strains, as those that carry the SCC*mec* cassette type IV, are susceptible to zinc indicating that zinc resistance is not the only factor contributing to the spread of MRSA (Cavaco et al., 2011). Actually, similarly to tetracycline treatment, it has been reported that transmission of MRSA between positive and negative animals was not influenced by the short-term exposure to zinc-oxide (Moodley et al., 2011a). Interestingly, other metal resistance genes have been discovered recently in novel SCC*mec* cassette types IX and X among MRSA CC398 isolates recovered from participants at a veterinary conference in Denmark (Li et al., 2011). The SCC*mec* cassette type IX was found in an isolate from a Thai participant, while the SCC*mec* cassette type X was described in an isolate from a Canadian. Both SCC*mec* cassettes include copper (*copB* gene), cadmium (*cadDX* operon), and arsenate (*arsRBC* or *arsDARBC* operons) resistance elements (Li et al., 2011). So far, however, few studies determined susceptibility to copper sulfate in MRSA CC398 isolates from pig origin (Cavaco et al., 2011). Moreover, Cavaco et al. (2011) reported no association between the minimal inhibitory concentrations (MICs) of copper sulfate and methicillin resistance. Both MRSA (20%) and MSSA (66%) isolates showed high levels of resistance to copper sulfate (MIC > 12 mM) (Cavaco et al., 2011). Still, copper sulfate resistance has been described in other gram-positive livestock-associated bacteria (i.e. enterococci) (Aarestrup and Hasman, 2004). Given the few reports, further research should be done to establish the prevalence of metal resistance genes other than *czrC* among LA-MRSA as well as the possible role of these metals in its dissemination among pigs.

## Transmission of LA-MRSA

### Transmission from pigs to pigs

**Figure 1:**
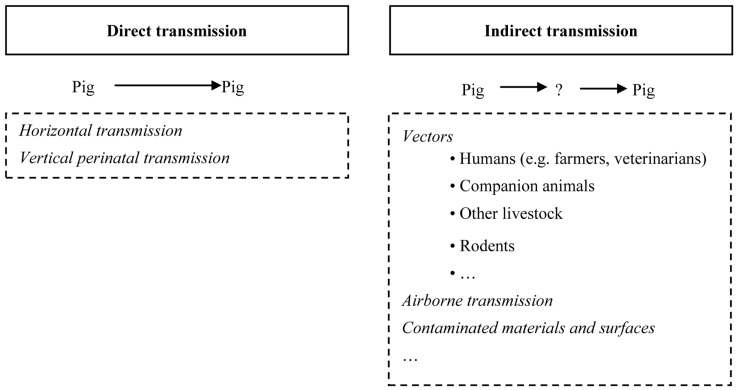
**Schematic overview of the potential transmission routes of MRSA between pigs**.

Transmission between hosts is a critical feature in the epidemiology of any pathogen (Massey et al., 2006). Since pigs have been recognized as important reservoir of LA-MRSA, studies have been done to determine the within- and between-herd transmission routes. From these studies it appeared that MRSA can be transmitted among pigs by direct and indirect contact (Figure 1).

#### Direct transmission

Transmission by direct contact is probably the main route for MRSA transmission between pigs (Broens et al., 2012a,b). Indeed, it has been suggested that MRSA-positive pigs might play a crucial role in transmission of these bacteria to negative animals (horizontal transmission) (Broens et al., 2011c,d). In that way, only a few positive animals can result in propagation of MRSA on farms or even beyond farms, through purchase of MRSA-positive pigs (van Duijkeren et al., 2008; Espinosa-Gongora et al., 2012). Additionally, some studies suggested MRSA transmission between pigs in slaughterhouses due to the high density of the animals during the housing in the abattoir (de Neeling et al., 2007; Tenhagen et al., 2009; Broens et al., 2011d). Broens et al. (2011d) reported that MRSA-negative pigs became MRSA-positive within a short time during transport to the abattoir, going from 0 to 10.3% (12/117), and at stunning, 59.8% (70/117) of these animals were positive. Nevertheless, transmission by indirect contact appeared to play an additional role since 43.3% of the negative animals from a single batch were positive at stunning, even without contact with other batches.

MRSA can also be transmitted from sows to their offspring (vertical perinatal transmission) (Espinosa-Gongora et al., 2011; Weese et al., 2011; Broens et al., 2012a; Verhegghe et al., 2012a). In fact, in an experimental study, transmission of MRSA from a sow to all newborn piglets has been demonstrated, representing the effectiveness of vertical perinatal transmission (Moodley et al., 2011b). On top of this, some studies (Weese et al., 2011; Verhegghe et al., 2012a) reported that piglets from MRSA-positive sows were more likely to be MRSA-positive. Still, MRSA was also reported in piglets from negative sows, indicating that other factors might additionally be involved. In this context, Verhegghe et al. (2012a) recently reported differences in MRSA colonization trends between different farrow-to-finish farms (i.e., low colonization vs. high colonization farms). In this study, each farm could be considered as a closed system in which different factors (such as environmental contamination) might play a role in the colonization of animals. Moreover, piglets either appeared to be intermittent carriers or were recolonized over time. Consequently, the sow’s colonization status is important and should be considered when implementing control measures. However, differences in colonization percentages between farms complicate the standardization of hygienic measures and require well-adapted control measures on each farm.

#### Indirect transmission

Humans have been shown to be susceptible to colonization/contamination with LA-MRSA (see next section), therefore, it is likely that persons in contact with pigs act as vectors, transmitting MRSA while handling the animals (within-herd dynamics) or introducing MRSA in negative farms in the case of veterinarians (between-herd dynamics).

Companion animals (cats and dogs) are commonly recognized as sources and vectors for recurrent MRSA colonization or infection of their human contacts (Manian, 2003; Sing et al., 2008; Denis et al., 2009; Loeffler and Lloyd, 2010). Generally, MRSA strains isolated from these animals have a human genetic background. However, LA-MRSA CC398 has occasionally been detected in cats and dogs due to transmission from humans (mainly veterinary personnel) (Witte et al., 2007; Nienhoff et al., 2009; Floras et al., 2010; Haenni et al., 2012). Until now, the prevalence of MRSA CC398 in companion animals residing on farms is unknown. Yet, Denis et al. (2009) reported positive MRSA carriage in dogs living in pigs farms. Moreover, Pletinckx et al. (2012) reported that cats and dogs living on a LA-MRSA-positive pig farm carried MRSA isolates related to those of the pigs living on the farm. Accordingly, companion animals residing on the farm may act as vectors, transporting MRSA from one area of the farm to another (within-herd dynamics) (Pletinckx et al., 2012). It was also remarkable that other “domesticated” animals (e.g., goats) residing on the farm appeared to carry related MRSA strains, even without direct contact with pigs (Pletinckx et al., 2012). So far, the role of other farm animals (e.g., poultry, cattle, and horses), on mixed farms, as a source of MRSA carriage in pigs remains largely unknown. Poultry and cattle appear to carry MRSA, though with a lower prevalence compared to pigs residing on the farm, and might therefore also play a role in the dissemination of MRSA on the farm (Pletinckx et al., 2011, 2012; Verhegghe et al., 2012b). Presently, few studies investigated the MRSA CC398 carriage rates in poultry (Nemati et al., 2008; Persoons et al., 2009; Mulders et al., 2010; Monecke et al., 2013). In Belgium, 12% (10/81) of *S. aureus* isolates from healthy chickens on 5 out of 39 sampled farms were found to be MRSA CC398 (Nemati et al., 2008). In another study, Persoons et al. (2009) reported a MRSA CC398 carriage rate of 10.7% (8/75) within 14.3% (2/14) of the investigated broiler farms. Moreover, MRSA CC398 was not detected in laying hens (Persoons et al., 2009). Similarly, in the Netherlands, MRSA CC398 was detected among 6.9% (28/405) broilers originating from 40 Dutch slaughter flocks of which 35% were positive (Mulders et al., 2010). Though MRSA CC398 has been found in poultry isolates, the majority of isolates reported, in both diseased and healthy chickens, belonged to the CC5 (Monecke et al., 2013), which is also one of the most successful human-associated lineages (Lowder et al., 2009). Concerning horses, MRSA CC398 has mainly been reported in equine clinics (Cuny et al., 2008; Hermans et al., 2008; Van den Eede et al., 2009; Sieber et al., 2011) but limited data is available at farm level (Van den Eede et al., 2012, 2013). In West-European horses admitted to a Belgian veterinary clinic, a MRSA CC398 carriage rate of 10.9% (12/110) was found (Van den Eede et al., 2009). However, Van den Eede et al. (2012) recently reported a low prevalence (0.53%) at farm level in Belgium. Similarly, very low and even absent farm level carriage rates of MRSA CC398 have been reported in the Netherlands (Busscher et al., 2006), Slovenia (Vengust et al., 2006), and Atlantic Canada (Burton et al., 2008). Healthy carriage of MRSA CC398 has also occasionally been reported in bovines. Carriage rates among veal calves have been reported ranging from 1% in Switzerland (Huber et al., 2010), 6.5% in France (Haenni et al., 2011) to 28–50% in the Netherlands (Mooij et al., 2007; Graveland et al., 2009, 2010). In Germany, MRSA CC398 was detected in nasal samples of dairy cows and calves on a farm where also pigs were raised and where MRSA was also found in mastitis milk samples (Spohr et al., 2011). Also in mastitis, MRSA CC398 has been reported in Switzerland (Huber et al., 2010; Sakwinska et al., 2011), Germany (Monecke et al., 2007; Feßler et al., 2010), and Belgium (Vanderhaeghen et al., 2010). In Switzerland, MRSA CC398 accounted for 1.4% (2/142) of the *S. aureus* strains from mastitis milk samples (Huber et al., 2010). In Germany, within-herd prevalences of MRSA CC398-positive cows were found to vary between 1.4 and 16.7% in three dairy farms (Spohr et al., 2011). In Belgium, a high prevalence of MRSA cases of subclinical and clinical mastitis in cows has been reported (Vanderhaeghen et al., 2010). Particularly, mastitis caused by MRSA CC398 was detected in 10% of tested Belgian farms (Vanderhaeghen et al., 2010).

Rodents are recognized for their role in transmission and persistence of zoonotic bacteria on livestock farms (Meerburg et al., 2006). van de Giessen et al. (2009) reported MRSA CC398 for the first time in black rats (*Rattus rattus*) living on pig farms. Later on, Pletinckx et al. (2012) demonstrated that 70.6% (12/17) of the black rats (*Rattus rattus*) and voles (*Microtus arvalis*) caught on four MRSA-positive farms carried MRSA CC398. Obviously, rodents may easily be contaminated by direct contact with contaminated feces, dust or by inhalation when roaming around in MRSA-positive stables. From then on, they can transport MRSA to other pig units (within-herd dynamics) or even beyond farms (between-herd dynamics).

As mentioned previously, the role of the environment in the spread of MRSA might be underestimated. Several studies reported MRSA-positive environments in association with MRSA-positive pigs [European Food Safety Authority (EFSA), 2009; van den Broek et al., 2009; Espinosa-Gongora et al., 2012; Friese et al., 2012; Pletinckx et al., 2012; Verhegghe et al., 2012a]. Furthermore, though cleaning and disinfection procedures are used, MRSA might survive in the environment and remain a source of contamination for newly introduced negative animals (Broens et al., 2011d). Moreover, LA-MRSA might be introduced by contaminated feed or material entering the pig units (Amass et al., 2006).

Finally, MRSA has been reported in air samples on several pig farms (Dewaele et al., 2011; Friese et al., 2012; Pletinckx et al., 2012; Verhegghe et al., 2012a). Apart of direct contact, airborne transmission is also a possible transmission route of MRSA between humans as has been reported in hospitals (Eames et al., 2009). Therefore, it is likely to occur between pigs within a farm. Moreover, airborne transmission might play a role in dissemination of MRSA between herds in close proximity to each other. Antimicrobial resistant *S. aureus* has been recovered outside pig facilities to at least 150 m downwind (Gibbs et al., 2006; Schulz et al., 2012). At present, however, the relative contribution of indirect transmission routes needs further investigation.

### Transmission from pigs to humans

Numerous studies reported that persons living or working on pig farms, including farmers and their family members, veterinarians, and slaughterhouse workers, are at increased risk for being colonized or infected with LA-MRSA (Voss et al., 2005; Lewis et al., 2008; van Duijkeren et al., 2008; Denis et al., 2009; van den Broek et al., 2009; Huber et al., 2010; Mulders et al., 2010; van Cleef et al., 2010a,b; Bisdorff et al., 2011; Garcia-Graells et al., 2012). In Belgium, 37.8% (48/127) persons working or living on 25 out of the 49 farms investigated carried MRSA CC398 (Denis et al., 2009). Another study reported that 9.5% (14/146) of the Belgian veterinarians who participated in this survey carried MRSA, of which 7.5% (11/146) belonged to MRSA CC398 (Garcia-Graells et al., 2012). Moreover, van Cleef et al. (2010a) reported that 5.6% (14/249) of the slaughterhouse workers enrolled in this study were MRSA-positive, which is significantly higher than the general prevalence in the Netherlands (0.1%) (Wertheim et al., 2004). Though exact transmission routes between pigs and humans have not been elucidated yet, it is likely that, similarly to pig-to-pig transmission, it occurs by direct and indirect contact (i.e., transmission by contaminated air or/and environment). Moreover, the presence of MRSA in humans is likely to be associated with the intensity of animal contact and with the within-herd MRSA prevalence (Meemken et al., 2008; Graveland et al., 2010). Graveland et al. (2011) reported that the MRSA prevalence in veal calf farmers and their family members decreased from 26 to 11% in absence of animal-exposure (−58%), with only 7% (11/155) of persistent carriers, suggesting that MRSA CC398 is a poor persistent colonizer in most humans. In addition, van Cleef et al. (2012) reported that, after short occupational exposure to MRSA-positive pigs or veal calves, MRSA was detected among 17% (34/199) of the field workers but within 24 h 94% (31/34) was again free of MRSA while the others became negative shortly thereafter. In contrast, Köck et al. (2012) reported that 59% (16/27) of previous MRSA-positive farmers did not clear their MRSA during summer leave, concluding that the absence from pig contact during the summer leave did not have an impact on MRSA colonization of pig farmers. Therefore, further studies are necessary to determine the capacities of LA-MRSA CC398 to persistently colonize humans.

### Transmission between humans

Presently, LA-MRSA has infrequently been reported beyond animal-exposed communities (van Loo et al., 2007; Cuny et al., 2009; Golding et al., 2010; van Cleef et al., 2010b, 2011; Wulf et al., 2012). Some studies examined human-to-human transmission of MRSA CC398 in hospital settings. Based on observational data, Wassenberg et al. (2011) reported that the relative nosocomial transmission risk for MRSA ST398 was 0.28 compared to non-MRSA ST398 genotypes. In an additional study, it appeared that MRSA ST398 was six times less transmissible compared to non-MRSA ST398 genotypes in Dutch hospitals (Bootsma et al., 2011). Moreover, recent genome sequencing data has suggested that the CC398 lineage originated from humans and later spread to livestock, and that this jump from human to animals was followed by a decreased capacity of human colonization, transmission, and virulence (Price et al., 2012). Still, the need to evaluate the transmission risk outside the hospital setting (i.e., in the healthy community) remains.

As for pigs, LA-MRSA ST398 does not appear to be highly infectious for humans, although reports on skin infections with MRSA have occasionally been published (Yao et al., 2010), as well as reports on more serious infections such as deep abscesses, cellulites, necrotizing fasciitis (Pan et al., 2009; Soavi et al., 2010), bacteremia (van Belkum et al., 2008; van der Mee-Marquet et al., 2011), and endocarditis (Ekkelenkamp et al., 2006). However, these data have to be interpreted with caution due to the over interest in such infections. During a wide EU surveillance, Grundmann et al. (2010) could not find any MRSA ST398, but a low prevalence of MSSA ST398 was found. So, infections with MRSA ST398 seem to be rare, but one should remain vigilant.

Albeit the increase in MRSA prevalence among (animal-exposed) humans, hospital outbreaks caused by LA-MRSA have only been occasionally reported so far (Witte et al., 2007; van Rijen et al., 2008, 2009; Wulf et al., 2008b; Pan et al., 2009; Schijffelen et al., 2010). In a Dutch hospital, van Rijen et al. (2008) reported that 13% (3/23) of the patients with MRSA not typeable by PFGE had an active infection caused by MRSA compared to 42% (21/50) of the patients with MRSA typeable by PFGE, suggesting a lower virulence of LA-MRSA. This is in accordance with the fact that, up till now, few virulence determinants have been detected among LA-MRSA CC398 (van Belkum et al., 2008; Welinder-Olsson et al., 2008; Yu et al., 2008; Kadlec et al., 2009; Walther et al., 2009; Salmenlinna et al., 2010; Schijffelen et al., 2010; Stegger et al., 2010; Argudín et al., 2011; Hallin et al., 2011; Monecke et al., 2011). However, a continuous surveillance of its epidemiology and virulence determinants is warranted, since some CC398 isolates with important human virulence factors as the bicomponent Panton-Valentine leukocidin (van Belkum et al., 2008; Welinder-Olsson et al., 2008; Yu et al., 2008; Salmenlinna et al., 2010; Stegger et al., 2010) or staphylococcal enterotoxins (Kadlec et al., 2009; Argudín et al., 2011) have been reported. Moreover, livestock-associated CC398 has been linked to an increase in MRSA infection in northern Europe (van Cleef et al., 2011).

## Pig Models to Study Colonization and Transmission Dynamics

At present, a number of experimental colonization models have been developed in order to understand colonization and transmission dynamics of LA-MRSA among pigs, with the aim of future use for development of intervention strategies (Table 2) (Moodley et al., 2011b; Broens et al., 2012a; Crombé et al., 2012b; Jouy et al., 2012; Szabó et al., 2012). The experimental design (i.e., inoculation strategy, type and dose of inoculum, age of the animals, strain, and use of antibiotics), the laboratory techniques as well as the pre-defined criteria for colonization of these models differ largely which makes comparison difficult.

**Table 2:** **Experimental MRSA colonization models**.

Factor	Inoculation model						
	Nasal-gastrointestinal	Vaginal	Nasal	Oral	Nasal	Nasal	Nasal-skin
Type of inoculum	ST398/t011, t034, t108 and ST9/t899		First: MSSA ST9 t337, Second: MRSA ST398/t011	MRSA ST398/t011	MRSA ST398/t011	MRSA ST398/t011 (4 concentrations)	MRSA ST398/t011
Dose of inoculum	∼1 × 10^8^ CFU/nostril, ∼1.5 × 10^9^ CFU/stomach	∼4.5 × 10^9^ CFU over 3 days	First: ∼2 × 10^8^ CFU, Second: ∼2 × 10^8^ CFU	∼5 × 10^9^ CFU	∼2 × 10^4^ CFU	A: ∼5 × 10^2 ^CFU, B: ∼5 × 10^4^ CFU, C: ∼5 × 10^7^ CFU, D: ∼5 × 10^8^ CFU	∼3 × 10^8^ CFU/nostril, ∼1.5 × 108 CFU/skin behind the ear
Age of inoculation	6-week-old SPF	A 97-day pregnant SPF sow	First: 7-week-old, Second: 9-week-old	7-week-old	8-week-old SPF	4-week-old	3-week-old
Experimental period	23 days	28 days	55 days	15 days	21 days	14 days	43 days
Antimicrobial treatment	1-week tetracycline	18 days tetracycline	Absent	Absent	Absent	NS	Absent
Housing	Grouped		Individually	Grouped	Grouped	NS	Grouped
Diagnostic tool	Nares and rectum	Sow: nares, teats, inner vagina, perineum, Piglets: nares, mouth, rectum	Nares, rectum, and vagina	Nares and rectum	Nares and feces	Skin, nasal mucosa, and conjunctiva and in feces	Nares, skin, perineum, throat, and environment (wall and feeder)
Time of diagnostic	1×/week		Every 2 days		Every 2 days	Daily for First 3 DPI; 2×/week	Every 2 days
Definition of colonization	Four MRSA + cultures over a period of 4 weeks		NS		NS	>50% MRSA+ animals and MRSA+ inner organs on 21 DPI	NS
Conclusion	Unstable carriage	Stable colonization; sow developed metritis	Unsatisfactory results; 2/5 animals sporadically MRSA+	Stable colonization; 4/5 piglets died	Sporadic MRSA detection; MRSA+ lymph nodes	Effective colonization with dose D	Successful transmission, stable colonization, Environment as supplementary source
Reference	Moodley et al. (2011b)	Moodley et al. (2011b)	Broens et al. (2012a)	Broens et al. (2012a)	Jouy et al. (2012)	Szabó et al. (2012)	Crombé et al. (2012b)

*CFU, colony forming unit; SPF, specific pathogen free; MRSA+, methicillin-resistant S. aureus positive; NS, not specified; DPI, days post inoculation*.

Nasal and nasal-gastrointestinal inoculation with LA-MRSA has been found not to result in successful colonization of piglets in some studies (Moodley et al., 2011b; Broens et al., 2012a), while others achieved colonization using relatively high bacterial inoculum doses (Broens et al., 2012a; Crombé et al., 2012b; Szabó et al., 2012). Unfortunately, in the study of Broens et al. (2012a), this high dose led to the development of lethal necrotizing pneumonia in 80% (4/5) of the inoculated animals, making this procedure unsuitable for future experiments. In contrast, Szabó et al. (2012) reported colonization without any clinical implications though MRSA was detected in the inner organs (i.e., palatine tonsils, mandibular lymph nodes, spleen, lung, and ileocecal lymph nodes), 21 days after inoculation (Szabó et al., 2012). Interestingly in this context is that even if a low bacterial inoculum dose does not result in effective colonization, MRSA can be detected in the tonsils and the lymph nodes draining the neck (i.e., axillary, retropharyngeal, and cervical lymph nodes) (Jouy et al., 2012). It remains however unclear why high inoculum doses are needed to get individual animals colonized. It may be hypothesized that *in vitro* culture does not create the best physiological state of the bacterium, while this is the case when the bacteria underwent an animal passage.

Moodley et al. (2011b) described an intra-vaginal inoculation method which, in contrast to their first nasal-gastrointestinal model, was successful and resulted in stable colonization of the sow and her piglets after farrowing. However, from a practical point of view, this vaginal inoculation model is not evident due to the time-consuming aspect and the health risk of the method, illustrated by the development of lethal metritis in one of the sows. Consequently, these experimental colonization studies pointed out the complexity of colonization with LA-MRSA. Both host-associated and environmental factors could influence this inherent variation in susceptibility to MRSA carriage (Moodley et al., 2011b; Broens et al., 2012a; Crombé et al., 2012b; Szabó et al., 2012).

Using these experimental colonization models, transmission experiments were performed by our team (Crombé et al., 2012b) and the team of Broens et al. (2012a) to quantify the spread of MRSA among weaned piglets. In our study, shortly after introduction of MRSA ST398, transmission occurred, indicating a very rapid spread (Crombé et al., 2012b). Another observation is that, depending on the animal, carriage may be intermittent, suggesting that animals recover without immunity and thus become susceptible again. Therefore, to assess the transmission rate of MRSA among weaned piglets, transmission was assumed to be in accordance with a susceptible-infectious-susceptible (SIS) model (Velthuis et al., 2003). The basic reproduction ratio (*R*_0_), which characterizes transmission, estimated based upon these experiments varied between 3.92 and 52.54. If the *R*_0_ is above 1, it means that a typical infectious animal generates more than one secondary case and that the agent is likely to persist in the population (Velthuis et al., 2003, 2007). Our observations are in accordance with the other recent study of Broens et al. (2012a) whom also investigated the transmission potential of MRSA ST398 though with different methodologies. Broens et al. (2012a) reported a *R*_0_ that varied between 3.7 and 4.3. The results obtained in these studies therefore suggest that, after introduction, MRSA ST398 can easily spread among animals (despite the absence of antimicrobial usage), with a tendency to become established.

## Control Measures

**Figure 2:**
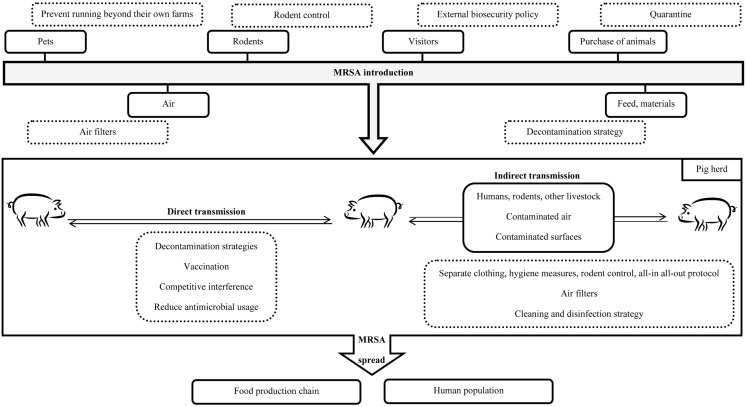
**Schematic representation of possible MRSA transmission routes (black line) and theoretical intervention strategies (pointed gray)**.

As presented in Figure 2, various prevention and intervention strategies have currently been suggested. Yet, the question remains open whether strict and extensive (sometimes costly and laborious) biosecurity procedures should be implemented for MRSA control knowing that, for now, the clinical relevance of MRSA CC398 remains minor (Meemken et al., 2010; van der Wolf et al., 2012). Also, if implemented, the effectiveness of the measures still needs to be certified since common measures such as washing (spraying with water, soaping, and rinsing with water) are not likely to affect the presence of MRSA on the skin of sows in MRSA-positive farms (Verhegghe et al., 2011).

It has been stated that reducing the antimicrobial use could reduce the number of colonized animals and/or lower the MRSA load shed by these animals (Broens et al., 2012b). Indeed, as mentioned previously, Broens et al. (2012b) recently reported higher transmission rates when tetracycline and β-lactams were used. Furthermore, Moodley et al. (2011a) showed that feed supplemented with tetracycline appeared to increase the nasal MRSA CC398 load of piglets in an experimental study but without influencing the transmission of MRSA to MRSA-negative animals. These findings might be explained by a selective advantage of MRSA CC398 compared to susceptible strains in the presence of these antimicrobials. However, several studies reported MRSA transmission even in absence of antimicrobial use (Weese et al., 2011; Broens et al., 2012a; Crombé et al., 2012b). So, antimicrobial use might have some influence on the MRSA load and/or predispose animals to MRSA colonization but it does not appear to be essential for MRSA acquisition and transmission. This suggests that reducing antimicrobial usage alone will not be sufficient to eradicate MRSA from a pig herd.

In light of MRSA CC398 control measures, it has been suggested that a *S. aureus* vaccine might help to reduce the number of colonized animals by inducing immunity and thus preventing colonization (Fluit, 2012). In murine animal models, antibodies directed against microbial surface components recognizing adhesive matrix molecules, immune-modulating proteins, and toxins were found to protect against nasal colonization and infection (Holtfreter et al., 2010). However, in humans, doubts on the feasibility of effective immunization against *S. aureus* have already been raised. Indeed, none of the anti-staphylococcal vaccines have successfully passed clinical trials up to now (Verkaik et al., 2011). Besides, it was found that colonization with *S. aureus* elicited a significant antibody response in persistent carriers compared to non-carriers or intermittent carriers, however, this does not seem sufficient to eliminate *S. aureus* (van Belkum et al., 2009; Verkaik et al., 2009). Moreover, *S. aureus* carriers still appeared to be susceptible to *S. aureus* infection despite the presence of high anti-staphylococcal antibodies. In fact, it is known that *S. aureus* carriers have a higher risk for developing infections compared to non-carriers (Wertheim et al., 2005). As in humans, it might be hard to induce immunity to *S. aureus* (mainly LA-MRSA) in pigs by vaccination knowing that, as mentioned previously, LA-MRSA does typically not induce disease. Actually, a recent study (Crombé et al., 2013) reported that a LA-MRSA isolate (ST398, *spa* type t011, SCC*mec* type V) did not elicit a significant humoral immune response in recently weaned, conventionally raised pigs, suggesting a low immunogenic potential of this strain in this age group during colonization. The asymptomatic carriage of this isolate induced an increase of immunoglobulin (Ig) G levels directed against staphylococcal microbial surface components recognizing adhesive matrix molecules (MSCRAMMs), which are known to play a role in *S. aureus* colonization. In contrast, IgG levels directed against staphylococcal toxins or immune-modulating proteins decreased over time, suggesting absence of bacterial invasion (Crombé et al., 2013).

Since the humoral immune response alone appears to be inadequate to eliminate *S. aureus* in humans, the role of the cellular immune response in protection against *S. aureus* needs to be considered. Recently, Arlian and Tinker (2011) developed a vaccine that combines a *S. aureus* antigen and an adjuvant, respectively IsdA (which is important in *S. aureus* colonization) and cholera toxin (CT) A2/B (a potent immunostimulatory molecule that binds to and targets effector cells to the mucosal site). Intranasal administration of this IsdA-CTA2/B chimera in mice induced systemic IgG, mucosal IgA, and cell-mediated response. The question still remains whether this mucosal vaccine will lead to reducing or even preventing *S. aureus* colonization in humans. It will be challenging for future research.

Further measures should be investigated, while meanwhile, transmission between positive and negative farms should be avoided. Therefore, it is advisable that farms check upon their MRSA status and if negative, they should only purchase animals from negative farms and take extreme care of vectors that may introduce MRSA in their farm.

## Perspectives

Research performed so far indicates that MRSA CC398 has a high transmission potential and also a high probability to persist in the pig population at intensive farming. Moreover, these bacteria survive in the pig environment, which might furthermore act as an additional source of MRSA contamination. The control or eradication of MRSA CC398 from pig herds with intensive farming procedures can therefore be expected to be very difficult. Furthermore, the fact that MRSA CC398 currently appears as having a minor impact on pig health, questions the need of implementing expensive national eradication/control programs. Yet, the ability of MRSA CC398 to transfer from pigs (and other livestock) to humans is known as one of the primary concerns of its emergence. Indeed, persons in close contact with pigs are at higher risk to be colonized with MRSA CC398 (Voss et al., 2005; Denis et al., 2009; Mulders et al., 2010; van Cleef et al., 2010a,b). Still (human), infections and outbreaks are infrequently reported at the moment (Witte et al., 2007; van Rijen et al., 2008, 2009; Wulf et al., 2008b; Pan et al., 2009; Golding et al., 2010; Grundmann et al., 2010; Schijffelen et al., 2010). Moreover, it has been shown that MRSA CC398 has lower nosocomial transmissibility (Bootsma et al., 2011; Wassenberg et al., 2011) and virulence compared to other (hospital-associated) MRSA clones (van Rijen et al., 2008, 2009). Considering this, MRSA CC398 appears to be a less important public health threat compared to human-associated genotypes. Still, the ability of MRSA CC398 to acquire virulence and resistance genes incorporates the potential adaptation into more virulent strains. More importantly, these animal-associated bacteria are a reservoir of resistance genes (e.g., *cfr* gene, which confers resistance to five different antimicrobial classes, including oxazolidinones such as linezolid, one of the few active drugs used for treating MRSA in human medicine and the *vga* genes, which as *cfr*, confer resistance to pleuromutilins also used in human and veterinary medicine (Kadlec et al., 2012) and might act as donor of these genes for HA-MRSA and CA-MRSA clones, which could result in a major public health burden due to potential treatment failure (Vandendriessche et al., 2011b)). Hence, while eradication programs face a high potential for failure, it might be wiser to continuously survey the evolution (including the presence of virulence and resistance genes) of animal-associated MRSA among pigs (and other livestock).

## Conflict of Interest Statement

The authors declare that the research was conducted in the absence of any commercial or financial relationships that could be construed as a potential conflict of interest.
